# Construction of *Clostridium tyrobutyricum* strain and ionic membrane technology combination pattern for refinery final molasses recovery and butyric acid production

**DOI:** 10.3389/fmicb.2023.1065953

**Published:** 2023-02-07

**Authors:** Bing Wang, Xiang Zhou, Wei Liu, Mei-Han Liu, Dan Mo, Qing-Feng Wu, Ya-Juan Wang, Miao-Miao Zhang, Lei Chen, Shan Yuan, Bo Zhou, Xin Li, Dong Lu

**Affiliations:** ^1^Institute of Modern Physics, Chinese Academy of Sciences, Lanzhou, China; ^2^CAS Key Laboratory of Bio-Based Materials, Qingdao Institute of Bioenergy and Bioprocess Technology, Chinese Academy of Sciences, Qingdao, Shandong, China; ^3^University of Chinese Academy of Sciences, Chinese Academy of Sciences, Beijing, China; ^4^College of Food and Bioengineering, Henan University of Science and Technology, Luoyang, Henan, China; ^5^College of Food Science and Engineering, Central South University of Forestry and Technology, Changsha, China; ^6^Gansu Key Laboratory of Microbial Resources Exploitation and Application, Lanzhou, China

**Keywords:** *Clostridium tyrobutyricum*, refinery final molasses, ionic membrane, adaptive evolution, butyric acid

## Abstract

**Introduction:**

*Clostridium tyrobutyricum* has considerable prospect in the production of organic acids. Globally, refinery final molasses is rich in sugar and reported to have high levels of accumulation and high emission costs, recognized as an excellent substrate for *C. tyrobutyricum* fermentation, but there is no suitable method available at present.

**Methods:**

In this study, an acid-base treatment combined with a new green membrane treatment technology – a dynamic ion-exchange membrane -was used to pretreat refinery final molasses, so that it could be used for *C. tyrobutyricum* to produce butyric acid. A high-performance liquid chromatography method was established to determine the conversion of a large amount of sucrose into fermentable sugars (71.88 g/L glucose and 38.06 g/L fructose) in the treated refinery final molasses. The process of sequential filtration with 3, 1, and 0.45 μm-pore diameter dynamic ion-exchange membranes could remove impurities, pigments, and harmful substances from the refinery final molasses, and retain the fermentable sugar.

**Results and discussion:**

This means that refinery final molasses from the sugar industry could be utilized as a high-value by-product and used for the growth of *C. tyrobutyricum*, with industrial feasibility and economic competitiveness. Using the treated refinery final molasses as a carbon source, *C. tyrobutyricum* was screened by the method of adaptive evolution. The strain with butyric acid yielded 52.54 g/L, and the yield of the six carbon sugar was increased from 0.240 to 0.478 g/g. The results showed that combination of *C. tyrobutyricum* and ionic membrane technology broke through the bottleneck of its utilization of refinery final molasses. This study provided an innovative idea for the *C. tyrobutyricum* fermentation to produce butyric acid.

## 1. Introduction

Globally, sugarcane is the main crop utilized for sugar production, and the world’s main cash crop. Sugarcane is planted across approximately 26 million hectares in more than 100 countries around the world, providing 75–80% of global sugar production and 40% of ethanol, which is produced by using sugarcane as a raw material (Aung et al., 2022). Sugar production from sugarcane is mainly concentrated in Asia and South and Central America. The annual global sugar yield is about 180 million tons, and Brazil dominates among producing countries, followed by India, the European Union, and Thailand. It is estimated that 100 tons of sugarcane can produce 10–12 tons of white sugar, 30–34 tons of bagasse, 3–5 tons of filter mud, and up to 4–4.5 tons of refinery final molasses when processed in the factory (Srivastava, 2020). As global sugarcane production is increasing year by year, sugar production has greatly exceeded consumption, leading to oversupply (Zhu and Pan, 2022). Furthermore, the by-products of industrial sugar production have now accumulated to unprecedented levels. Therefore, the further exploitation of value-added products is an innovative move to realize the diversification of the sugarcane industry, which will help to alleviate some of the sugar industries thorny problems.

The intermediate product obtained by the raw sugar factory after centrifugation becomes molasses A. Generally, molasses A, accounts for approximately 12% of the raw sugar separated by centrifuge, and the rest is molasses B. The “first pressed” sugar crystal obtained from syrup crystallization is combined with B molasses to form C molasses. After boiling and centrifugation, the last part of the sugar is recovered, and the rest is referred to as refinery final molasses (Nunes et al., 2020). The average total sugar content of the refinery final molasses is approximately 50% (mainly sucrose with a small amount of glucose and fructose), containing approximately 4% of the protein, trace elements (calcium, magnesium, potassium, and iron), and biotin (vitamin H or B7) (Khaire et al., 2021), which can be an excellent substrate for microbial fermentation. Although refinery final molasses is rich in nutrition, it contains a large number of colloidal and coloring substances. Due to its complex components, only 15% of refinery final molasses is traded internationally. At present, refinery final molasses is only used to add to dry feed to improve its palatability, and may cause toxic harm to animals if fed incorrectly (Moore and Ming, 2011). To date, no economically feasible method has been identified to recover refinery final molasses. In recent years, the global annual output of refinery final molasses reached 55 million tons (Zhang et al., 2021). The accumulation of refinery final molasses not only increases the waste management costs of factories, but also causes serious environmental pollution problems due to the discharge of high color waste liquid. Therefore, this study is committed to identifying an advanced and low-cost green treatment process by which to turn refinery final molasses into treasure.

The separation of colloidal and colored substances in refinery final molasses is the key to its reuse by microorganisms. Traditional separation technologies such as chemical oxidation, physical adsorption (activated carbon, macroporous resin), and biodegradation (microbial and enzymatic hydrolysis) usually have the disadvantages of low efficiency, high cost, large levels of pollution, and the introduction of new impurities (Meghana and Shastri, 2020), so that they are not commonly used to treat molasses. While membrane separation technology such as the ionic membrane is more commercially attractive for industries due to its simple equipment, convenient operation, energy saving capacity, environmental protection, mild process, and lack of phase change. The ionic membrane as a precision filter material, stands out among the numerous membrane products by virtue of its significant advantages, such as good interception characteristics, easy cleaning, simple operation, and economic benefits. According to the national pharmacopoeia of the United States, Japan, South Korea, and the European Union, ionic membranes of various pore sizes are used on large infusion sets (Mahat et al., 2018). Ivleva et al. (2020) showed that ionic membranes could be used to separate colloids and remove pollutants from natural water, and thus purify the water. In view of this, it may be feasible to utilize ionic membranes for the clarification and decolorization of refinery final molasses.

Butyric acid is an important basic and fine chemical product, which is widely used in food, chemistry, medicine, and other fields. The annual trading volume is more than 80,000 tons, and the price is approximately USD$1.8 (€1.6) per kilogram, globally (Akhtar et al., 2020). At present, butyric acid is mainly produced by chemical synthesis in the industry. With the development of green concepts and the demand for bio-based organic acids in food and drugs, butyric acid produced from plant-based raw materials in microbial fermentation is favored. *Clostridium tyrobutyricum* is the most studied butyric acid-producing bacterium at present. Starch, cheese whey, lignocellulosic biomass, and algae biomass have been used to produce butyric acid. Jiang et al. (2009) showed that during the fed-batch fermentation in FBB, the maximum concentration of butyrate could be achieved using sulfuric acid treated molasses (55.2 g/L), which is the best pretreatment technique for molasses. Guo et al. (2020) successfully over-expressed sucrose metabolism genes (scrA, scrB, and scrK) for *C. acetobutyricum* ATCC 824 in *C. tyrobutyricum* ATCC 25755, and 45.71 g/L butyric acid was obtained by batch fed fermentation using untreated molasses as a carbon source. However, the fermentation of *C. tyrobutyricum* with refinery final molasses as a carbon source has not yet been investigated.

The cost of the raw materials and its treatment usually accounts for approximately 50% of the cost of the final products for bio-based commodity chemicals (Baroi et al., 2017), which is a key factor used to measure the economic benefit of fermentation production. Pure sugar or food-derived biomass is widely used in microbial fermentation, but there are some problems such as the high price of raw materials, grain competition, and the low level of economic benefit. Refinery final molasses is an industrial waste that is low cost, as it is approximately only 7–10% the price of glucose but has the same sugar content (Khaire et al., 2021). Lignocellulosic biomass is expensive to enzymatically hydrolyze, while in contrast there is no stubborn and refractory cellulose in refinery final molasses, which could also reduce the cost of the raw material treatment. When compared with cheese whey and algae biomass, refinery final molasses were found to have higher sugar content and fermentation rates. In addition, they also contain nitrogen sources, amino acids, vitamins, minerals, and other growth factors after being treated (Li et al., 2021), so there is no need to add other substances to the fermentation process, which further reduces the fermentation cost of *C. tyrobutyricum*. A simple, economical and environmentally-friendly ionic membrane was used to remove the colloids and coloring substances in refinery final molasses, to turn waste into treasure, and further improve the yield of butyric acid, making refinery final molasses a good choice for microbial fermentation and butyric acid production.

In conclusion, the utilization of the ionic membrane, which is a simple and green method, can make refinery final molasses available to microorganisms. While it has potential for global organic acid production and the value-added industry of sugarcane by-products, it also has important significance for the recovery and reuse of biological resources, the protection of the ecological environment, and the healthy and sustainable development of future economies. The industrial production of butyric acid from refinery final molasses is expected to be offered imminently as a treatment option.

## 2. Materials and methods

### 2.1. Treatment of refinery final molasses raw materials

One kilogram of refinery final molasses was weighed and mixed with distilled water at a ratio of 1:4. The pH value of nearly 5 L of the sugarcane molasses solution was adjusted to 3.5 with a 98% concentrated sulfuric acid solution, and heated in a water bath at 90°C for 2 h. After boiling, it was continuously stirred in a ventilated environment. When the refinery final molasses was cooled to 70°C, the pH value was adjusted to 6.5–6.8 with Ca(OH)_2_, and it was left to stand overnight for clarification. The clear supernatant was diluted with distilled water to a sugar content of approximately 60 g/L.

**FIGURE 1:**
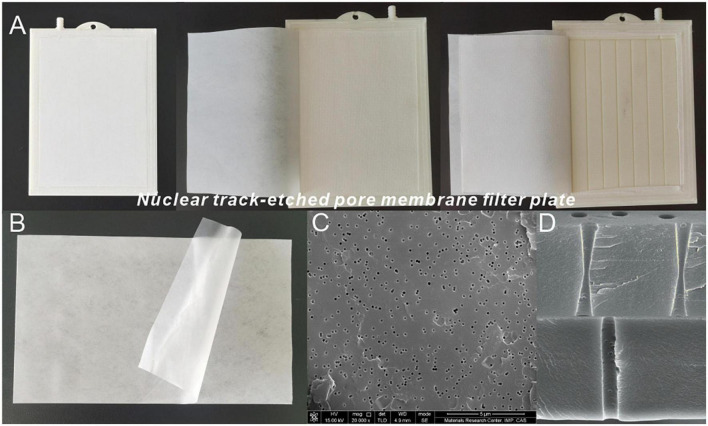
Ionic membrane and ionic membrane filtration machine. **(A)** Ionic membrane filter plate. **(B)** Ionic membrane element. **(C)** Scanning electron microscope (SEM) images of the ionic membrane from Modan’s article (Mo et al., 2014). **(D)** Cross sections of Positron Emission Tomography (PET) with pores displaying double cone shape and cylindrical shape from Modan’s article (Mo et al., 2014).

The refinery final molasses was filtered using ionic membranes with different interception pore sizes (Figures 1A, B) and ionic membrane filtration machines provided by the Materials Research Center of Institute of Modern Physics, Chinese Academy of Sciences. 2.5 L of the refinery final molasses after acid-base treatment was poured into the liquid tank, and the ionic membrane was fixed on the membrane connector. Aeration was turned on, and then the knob of liquid flowmeter was turned off to adjust the flow of the membrane liquid, and finally the coating contributing water valve was opened and the container was placed at the delivery port to collect the filtrate. The raw materials were treated using the above method, and the following 7 samples were prepared for subsequent experiments. Sample 1: refinery final molasses is mixed with distilled water at a ratio of 1:4; Sample 2: refinery final molasses after the acid-base treatment; Sample 3: refinery final molasses filtered by ionic membrane with 3, 1, and 0.45 μm pore sizes in turn.

### 2.2. Detection of the physical and chemical properties of different refinery final molasses

The Brix of the raw material was determined using the Refractometric Brix method. The pH of the feedstock was measured using a portable pH meter. The particle size distribution of the raw materials was measured using a laser particle size analyzer (Mastersizer, 2000, UK). The model of Sampler used is Hydro 2,000 MU (A) and the wet injection was adopted. The power was turned on and the instrument was preheated for 20–30 min. Then the cycle injector switch, computer and data processing software were turned on. Tap water was poured into the sample tank and cleaned ultrasonically three times. The refractive index, analysis mode, sampler, sampling rate, test times, and other parameters were set to test the background. A 25 mL sample to be tested was taken out and put into the beaker, and then the beaker was put into the ultrasonic cleaning machine to disperse the sample. Finally, the power was turned off and the beaker was removed. Finally, background measurements and sample measurements were completed by the machine.

The sucrose, glucose, fructose, maltose, and lactose content in the raw materials were determined using high performance liquid chromatography (HPLC) (Bruker, Germany) combined with a refractive index detector (RID). The mobile phase of the HPLC was 75% acetonitrile and 25% ultrapure water. The chromatographic conditions were as follows, column temperature: 30°C; detector: 45°C; injection volume: 15.0 μL; flow rate: 1 mL/min; running time: 30 min. An Aminex^®^ HPX-87P sugar analysis column was washed with 75% acetonitrile for 1 h before injection. Standard samples with gradient concentrations of five sugars were prepared and filtered by membrane filters (0.22 μm, Millipore), 1 mL of which was taken out and injected into a 1.5 mL chromatographic injection bottle for detection and analysis. According to the peak information for the five sugars, the standard curves were drawn. Refinery final molasses and fermentation broth samples were filtered by a 0.22 μm microporous membrane, and then sampled and analyzed.

### 2.3. Utilization of different refinery final molasses by *C. tyrobutyricum*

The *C. tyrobutyricum* ATCC 25755 was preserved in our laboratory. The reinforced *Clostridium* medium (RCM) for strain activation consisted of 50 g/L RCM medium and 3 g/L yeast extract. The refinery final molasses medium used the refinery final molasses solution instead of water as the solvent, which contained 3 g/L yeast extract and 10 g/L of the trace element solution. The trace element solution contained (g/100 mL): NaCl 0.1;MgCl_2_⋅6H_2_O 0.05;KH_2_PO_4_ 0.02;NH_4_Cl 0.03;KCl 0.03;CaCl_2_⋅2H_2_O 0.0015. If the above medium needed to be configured as a solid, 20 g/L agar had to be added. Before sterilization, the pH of the final medium was adjusted to 6.5–6.8 with HCl or NaOH. The carbon source was sterilized at 121°C for 20 min, and then aseptically added to the Petri dish, test tube, or fermentation bottle at room temperature (20–25°C). After inoculation, N_2_ was injected into the culture container for 2 min, and quickly sealed with a sealing film to ensure that the anaerobic environment was maintained. During the culture period, the syringe was used to regularly deflate the container to prevent it from exploding.

In the anaerobic incubator, an appropriate amount of *C. tyrobutyricum* bacterial solution stored at −80°C was dipped by the inoculation ring and scribed on the surface of the RCM agar plate, and then cultured at 37°C for 24–48 h. Single colonies were picked up and inoculated into an anaerobic test tube containing 10 mL RCM medium and cultured at 37°C for 72–96 h to obtain an activated bacterial solution. The activated bacterial solution was coated on the surface with four kinds of refinery final molasses agar plates treated with different treatments. Then they were cultured at 37°C for 24 h to observe the growth of *C. tyrobutyricum*.

### 2.4. Adaptive evolution and shake flask fermentation of *C. tyrobutyricum* using refinery final molasses as a carbon source

A single colony on the No. 4 refinery final molasses agar plate was picked and inoculated in an anaerobic test tube containing 10 mL of the refinery final molasses medium to obtain the first generation of seed solution. The above process for the solid-liquid co-culture with refinery final molasses as the culture medium was continued until the eighth seed solution was obtained and the adaptive evolution had occurred for a sufficient amount of time. The fermentation liquid in the 8th generation test tube culture was used as the seed liquid for the shake flask fermentation. After 36 h of fermentation 50 mL of the medium was added and the fermentation ended at 120 h. Samples were extracted after 24, 48, 72, 96, and 120 h respectively for analysis.

During adaptive evolution, *C. tyrobutyricum* was anaerobically cultured in a 37°C incubator for 48 h. During shake flask fermentation, *C. tyrobutyricum* was cultured at 37°C for 38 h, and then cultured in a shaker incubator at 37°C and 150 rpm for 120 h.

### 2.5. Detection of apoptosis

An Annexin V-FITC / PI apoptosis detection kit was used. The cells were collected into a centrifuge tube and centrifuged at 1,500 rpm for 5 min to remove the supernatant. The cells were then resuspended and counted with 1 mL 4°C pre cooled PBS, centrifuged at 1,500 rpm for 5 min, and the supernatant removed. Then 1 mL of 4°C pre cooled PBS was added to resuspend the cells, centrifuge at 1,500 rpm for 5 min, and the supernatant removed to obtain the cell sample. The binding buffer (4 mL binding buffer + 12 mL deionized water) was diluted with deionized water at 1:4. The cells were resuspended with 250 mL of the diluted binding buffer and the concentration was adjusted to 1 × 10^6^/mL. A 100 μL cell suspension was placed in a 5 mL flow tube, 5 μL annexin V-FITC was added and gently mixed. They were incubated in the dark at room temperature (20–25°C) for 10 min. Then 5 μL propidium iodide (PI) solution was added 5 min before the machine was put into operation, and gently mixed. Before using the machine, 400 μL PBS resuspended cells were added to the reaction tube and stored away from the light. Then flow cytometry (FACS) was performed. Annexin V-FITC showed green fluorescence and PI showed red fluorescence.

### 2.6. Detection of the physical and chemical properties of the fermentation broth

An appropriate amount of fermentation broth samples were taken out, and the OD value of the bacterial suspension was read at 600 nm with the enzyme labeling instrument (Bio Tek Epoch, USA). The sucrose, glucose, fructose, maltose, and lactose contents in the fermentation broth were determined by HPLC. The concentrations of butyric acid and acetic acid were determined by gas chromatography (GC) (456-GC, Bruker, Germany). The analysis conditions for GC were as follows: capillary column model: HP-INNOWAX, 30 m × 0.32 mm; detector (FID) temperature: 240°C; column temperature: 65–80°C, column flow: 3 mL/min; carrier gas (nitrogen) flow: 28 mL/min; gas (hydrogen) flow: 40 mL/min, auxiliary gas (air) flow: 300 mL/min; injection port temperature: 300°C; and the injection volume was 0.4 μL. Standard samples with gradient concentrations of the two acids were prepared, and after being filtered by a microporous membrane, 1 mL was removed and injected it into a 1.5 mL chromatographic injection bottle for detection and analysis. According to the peak information for the two acids, a standard curve was drawn. Fermentation broth samples were filtered using a 0.22 μm microporous membrane and then injected for detection and analysis.

All the above detection data were conducted three times in parallel, and the detection results were taken as the average value.

### 2.7. Statistical analysis

SPSS 17.0 (SPSS Inc, Chicago, IL) software was used to conduct two-factor random analysis of variance (two-way ANOVA) and minimum significant difference method (LSD) at *p* < 0.05. Origin 2021b (origin 2021b Inc, USA) was used to draw the curve chart, and the level of significance was set top = 0.05.

## 3. Results and discussion

### 3.1. Treatment of refinery final molasses by an ionic membrane providing carbon source for *C. tyrobutyricum*

The ionic membrane was made of thin-film polymer irradiated by a heavy ion beam using cyclotron technology (Mo et al., 2014). When combined with the ionic membrane filtration machine, it could also filter refinery final molasses. The working principle is that under the action of the static pressure difference, particles larger than the size of the membrane holes are blocked by the membrane, while smaller particles can pass through smoothly (Zhang et al., 2018). Under the action of certain pressure differences, a well-proportioned precoating agent solution could quickly coat the surface of the ionic membrane to form a filter cake layer with separation performance and the filter cake replaced the function of the membrane medium. During membrane filtration, the suspended refinery final molasses pollutants were intercepted or adsorbed on the ionic membrane surface under the action of pressure. When the filter cake filtration resistance reaches a certain value, backwashing and aeration scouring begin to clean both the prefabricated filter cake and the intercepted matter. The middle of the finished membrane element was a layer of an ABS plastic plate, and both sides were made of “two cloth and one film” composite membrane material which was an ionic membrane and polyester fiber non-woven fabric formed by ultrasonic welding or hot pressing (Figure 1A). In comparison with the original membrane thickness, it was found to further increase by 50–300%, resulting in super wear resistance, flexibility, and a supportive effect. The material of the ionic membrane is Positron Emission Tomography (PET) (Figure 1B). When observing the electron microscope image of the 0.45 μm pore size of the ionic membrane (Figure 1C), it could be seen that the membrane was a sieve filter material, with approximately cylindrical micropores, and the pore shape was round, and the size uniform. The pores of the dynamic ion films are prepared using a typical ion track etching method, which creates highly aligned nanochannels by selectively dissolving the potential tracks of the polymer films with chemical etchants (Mo et al., 2014). Using this method, the channel geometry can be accurately controlled by adjusting the chemical etching conditions, and the irradiated PET film can then etch the pore diameter corresponding to the pore density in the NaOH solution with a mass fraction of 15%. The pore shape in the ionic membrane was mostly regular, and commonly biconical or cylindrical to help minimize membrane pollution (Figure 1D). The cylindrical ionic membrane was selected to filter the refinery final molasses in this study. In the process of membrane separation, natural organic matter (proteins, polysaccharides, microorganisms, etc.) can cause organic pollution in the membrane leading to membrane surface adsorption, membrane pore blockage, and gel layer formation, which increases the cost and difficulty of membrane separation technology. In this study, the effective area of the cylindrical ionic membrane was 300–400 cm^2^. Even considering the most extreme use cases, the total filtration amount of the refinery final molasses of each membrane could reach 20–30 L without cleaning the membrane surface. In case of serious pollution, the total service time of the membrane after thoroughly cleaning the membrane surface with citric acid, vinegar, and detergent could also be restored to more than 80% of the original filtration performance (Zhang et al., 2018). Therefore, when compared with other membranes, the ionic membrane can reduce the membrane pollution caused by filtering refinery final molasses, maximize its use value, reduce costs, and thus have beneficial economic impacts.

Membrane separation technology is a new type of liquid separation and purification technology. Compared with traditional material separation processes, it can achieve separation and purification without introducing new components. Ionic membranes are classified as microfiltration membranes, which can penetrate solutions and 0.02–10.00 μm particles, and thus they can intercept micron and submicron fine suspended solids, microorganisms, and pollutants (Bogdanowicz et al., 2013). In addition to ionic, nanofiltration, ultrafiltration, and reverse osmosis the membranes can use different pressures to realize the selective separation of materials with different physical and chemical properties. Small molecules can pass through the nanofiltration membrane while they can retain small molecules with a mass fraction of 200–2,000 and dissolved components with a pore size of 1 nm. The ultrafiltration membrane penetrates solvent inorganic ions and smaller macromolecules of 0.001–0.020 μm, and intercepts colloids and macromolecules with a molecular with mass fraction greater than 500. The ionic membrane penetrates ions and trap solvents and macromolecule, while the reverse osmosis membrane permeability is 0.000–0.0010 μm for neutral small molecules, which means it intercepts suspended solids, macromolecules, and ions (Ghazali and Razak, 2021). Studies have shown that the macroporous adsorption resin method can separate organic macromolecules with specific polarity, such as flavonoids, alkaloids, and polysaccharides, from refinery final molasses, according to the principle of adsorption and molecular sieve (Guo et al., 2019). According to the investigation, the particle size of the macroporous resin, the diameter height ratio of the resin column, sample loading flow rate, elution flow rate, and other factors will affect its adsorption of effective components from refinery final molasses. Many factors need to be considered before application (Sen and Baidurah, 2021). In addition, compared with the complex pretreatment and regeneration process of macroporous resin, the ionic membrane has obvious advantages such as a simple operation and convenient cleaning, which is more conducive to the industrial treatment of refinery final molasses.

### 3.2. Effects of components of refinery final molasses on sugar transport of *C. tyrobutyricum*

The phosphoenolpyruvate carbohydrate phosphotransferase system (PTS) is the main pathway utilized for carbohydrate uptake by *C. tyrobutyricum* cells. Glucose and fructose in refinery final molasses are mainly phosphorylated by the PTS system located on and in the cell membrane, and they then enter the cell in the form of phosphorylated sugar. The PTS transport system of *C. tyrobutyricum* consists of two cytoplasmic proteins (enzyme I and heat stable protein HPR) and an enzyme complex related to sugar transport on the membrane surface (enzyme II). The two cytoplasmic proteins are encoded by genes PTS I and PTS h respectively. Enzyme II is a substrate specific protein complex composed of EIIA, eiib, EIIC, or EIID domains, at least one of which binds to the cell membrane of *C. tyrobutyricum*. EI obtains the phosphate group from the substrate of phosphoenolpyruvate, and then transfers the phosphate group to HPR. The phosphoryl group on the heat stable protein is catalyzed by EIIA and transferred to eiib, which is phosphorylated to activate EIIC. Sugars are phosphorylated by EIIC or EIID bound to the membrane, enter the cell membrane in the form of phosphorylated sugars, and enter the metabolic pathway of *C. tyrobutyricum* for absorption and utilization (Liu et al., 2020). During the acid production period of *C. tyrobutyricum* fermentation, the pH in the medium gradually decreases, which is not conducive to the environment for cell survival, leading to the start of sporulation and solvation. Cells form axial filaments through DNA replication, resulting in a decrease in the rate of DNA synthesis. The cells form an asymmetric septum at one end of the cell through asymmetric division, and the separated small part will form spores. Like phagocytosis, the formed septum surrounds the smaller part of cell division. Then, the spore cortex composed of peptidoglycan and a polypeptide chain is formed between the mother cell and developing spore. The cysteine rich protein forms a mantle protein, which contains more than half of the spore protein and develops into the presporum of the outer membrane protein. The cortical protein and spore sheath continued to develop and gradually matured, becoming a thin, white objects surrounding the presporium. Finally, the mother cell is lysed by lyase, and the spores released from the mother cell (Jiang et al., 2011).

**FIGURE 2:**
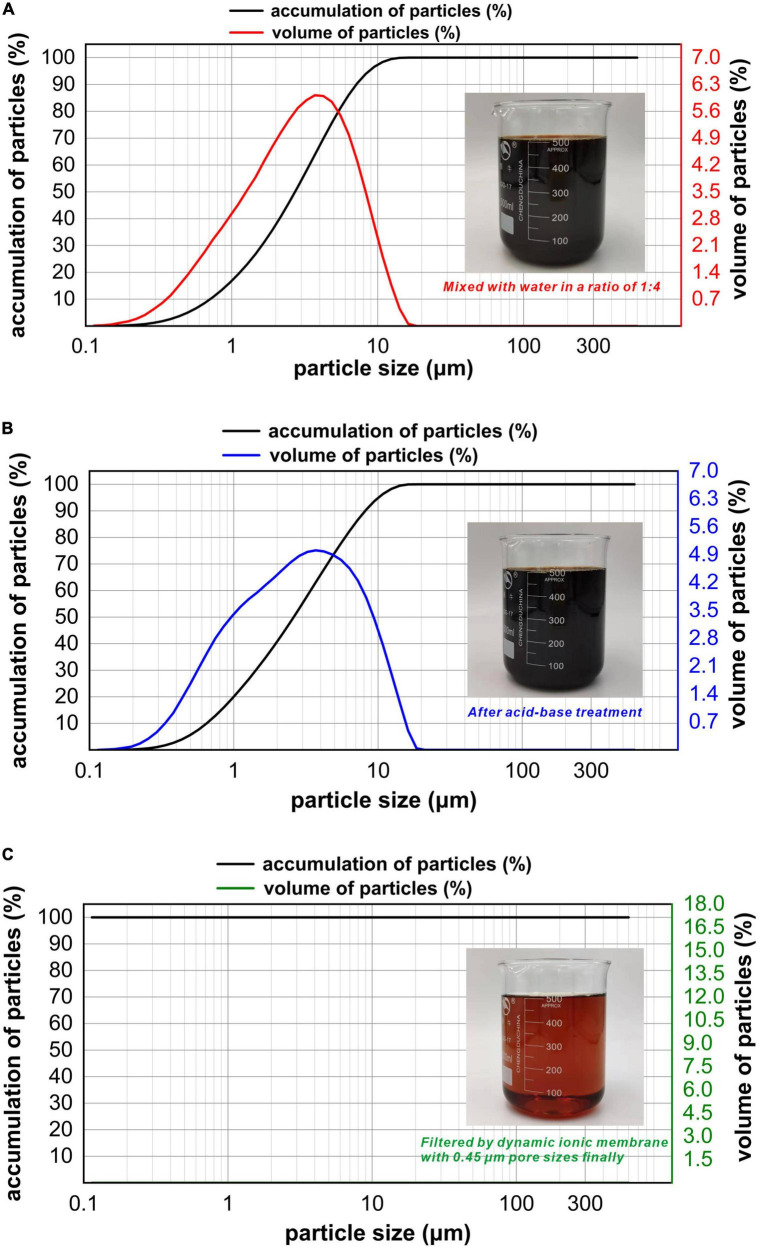
Particle size distribution plots for the refinery final molasses with different treatments. The measurement range is 0.02-2,000 μm, with water as the dispersion medium, injector model: Hydro 2000 MU (A), wet injection. When the laser irradiates the fine sample particles, scattering will occur, and the particle size distribution of the sample particles can be obtained using the relationship between the scattered light intensity and the number of particles of the corresponding particle size. The abscissa in the figure is the particle size of the particles in the solution, the abscissa is the partial size (μm), and the two ordinates are the accumulation of particles and volume of particles. **(A)** Particle size of the refinery final molasses diluted with water 1:4 and shown in a distribution diagram. **(B)** Particle size distribution diagram of refinery final molasses after acid-base treatment. **(C)** Particle size distribution diagram of refinery final molasses filtered with 3, 1, and 0.45 μm nuclear pore membrane in turn.

The particle size distribution of refinery final molasses before and after the acid-base treatment was compared and analyzed (Figures 2A, B). The particle size range of the refinery final molasses changed from 0–15 to 0–20 μm. The particle volume ratio of 0–0.114 μm particle size decreased from 0.01 to 0%, and the particle volume ratio of 0–0.973 μm particle size increased from 16.22 to 19.32%. This is because the acid-base treatment inevitably introduces new impurities into the process of converting sucrose into reducing sugar. Although dilute acid can effectively hydrolyze bagasse, aldehydes, phenols, and other substances unfavorable to cell growth will be produced more or less in the hydrolysis process (Fonseca et al., 2020). After hydrolysis, a large number of sodium ions will be produced in the process of neutralization with sodium hydroxide. The turbidity and chroma of the refinery final molasses treated by 0.45 μm ionic membrane changed obviously, and the particle size analysis showed that there were almost no particles larger than the pore size of the membrane (Figure 2C). The composition of refinery final molasses is complex (Wei et al., 2013), with sugars accounting for 46–52% of its content. Among them, the molecular weights of sucrose, glucose, fructose, and polysaccharide are 342, 180, 180, and > 500 Da, respectively. In addition, there is an abundance of pigments, macromolecular substances, organic acids, and inorganic salts. Pigments include caramel pigments with a molecular weight of < 50 kDa and polyphenols with a molecular weight of < 1,500 kDa. Macromolecular substances include pectin with a molecular weight > 100 kDa, deformed protein and dextran with molecular weights of approximately 100 kDa. Organic acids include aconitic acid (174 DA), citric acid (192 DA), and malic acid (134 DA). Inorganic salts are mainly sulfuric acid ash, including Na^+^, K^+^, Ca^2+^, Mg^2+^, and SO_4_^2–^, which are all < 100 kDa. Refinery final molasses contains high concentrations of phenols, nitrogen-containing heterocyclic compounds, polycyclic aromatic compounds, and other toxic and high content compounds, and most pigments and inorganic salts are difficult for microorganisms to utilize. The ionic membrane with pore sizes of 0.1–10 μm and operating pressures of 0.01–0.20 MPa can effectively intercept particles, colloids, viruses, and macromolecules. According to an investigation, into the sugar production process, due to enzymatic browning, non enzymatic browning, microbial activities, high temperature, the Maillard reaction, pre-ash treatments, and raw materials themselves, a large number of pigment non sugar components are produced, including phenolic pigments, caramel pigments, Maillard pigments, and hexose alkaline degradation pigments, which make the waste sugarcane sugar dark brown (Li et al., 2012). The ionic membrane can not only intercept suspended solids, remove a large amount of colloid in the solution, and clarify the solution, but also be used as an excellent medium for separating pigments from refinery final molasses.

### 3.3. Growth mechanism of *C. tyrobutyricum* in different treatments of refinery final molasses

**FIGURE 3:**
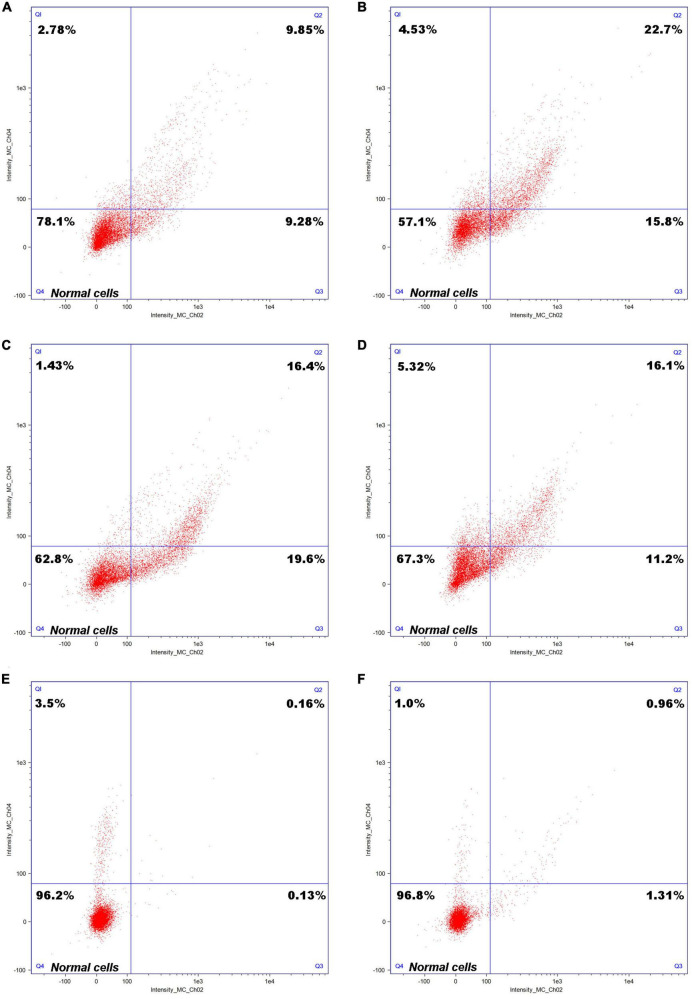
Cell staining of *Clostridium tyrobutyricum* with differently treated refinery final molasses samples. Cell staining of *C. tyrobutyricum* cultured to a logarithmic growth phase grown in refinery final molasses for **(A)** 6 h and **(B)** 12 h; after acid-base treatment for **(C)** 6 h and **(D)** 12 h; and after final treatment with 0.45 μm ionic membrane for **(E)** 6 h and **(F)** 12 h.

A cell staining diagram of the *C. tyrobutyricum* cultured to a logarithmic growth stage after growing in refinery final molasses, refinery final molasses after acid-base treatment, and refinery final molasses solution after ion-exchange membrane treatment for 6 and 12 h is shown in Figure 3. The Q4 region in the figure shows cells in good growth condition. Observing Figures 3A, B, *C*. *tyrobutyricum* was found to grow in refinery final molasses diluted with water only for 6 h, and the number of cells in the Q4 region accounted for 78.1% of the total number of cells obtained, which decreased to 57.1% after 12 h. The refinery final molasses mainly contains glucose, fructose, and sucrose, and the sugar content is sufficient. However, the components of the untreated sugarcane molasses are complex, and contain many stress factors that are not conducive to microbial growth, such as colloids, melanoids, and so on. The endocytosis of colloidal particles can increase the level of intracellular reactive oxygen species (ROS), thus changing the morphology, skeleton structure, adhesion and migration ability, proliferation ability, cell activity, gene expression, and other functions of the cells, causing serious damage to the *C. tyrobutyricum* cells (Huang J. et al., 2011). Melanoids are dark brown to black natural condensation products of sugars and amino acids. They are produced by a non enzymatic browning reaction called the Maillard reaction and are found in a large number of sugarcane molasses. Melanoids have complex structures, strong antioxidant activity and cytotoxicity (Satyawali and Balakrishnan, 2008). These damages are both dose-t and time-dependent. The stress factors in the refinery final molasses do not decrease during the growth of *C. tyrobutyricum*, but the number of *C. tyrobutyricum* decreases gradually with the increase of time. In addition, sugarcane molasses waste is acidic. Under acid-base stress conditions, it will inhibit the growth of production strains, interfere with cell metabolism, cause oxidative stress and ATP consumption, inhibit the glycolysis process, damage the stability of plasma membrane, and induce programmed cell death. The sucrose in the refinery final molasses accounts for approximately 50% of the total sugar. *C. tyrobutyricum* naturally has a relatively efficient ability to transport and metabolize glucose and fructose. Most *C. tyrobutyricum* do not have the ability to use sucrose. Therefore, the method of acid-base treatment was used to convert sucrose into a reducing sugar. According to Figures 3C, D, in the refinery final molasses after the acid-base treatment, the number of living cells growing for 6 h accounted for 62.8% (Q4) of the total number of cells, and the number of necrotic cells was 1.43% (Q1). Furthermore, the number of necrotic cells growing for 12 h was 5.32% (Q1). In the process for the acid-base treatment, some compounds such as organic acids, furan derivatives, and phenols, which inhibit the growth of microorganisms, were produced in sugarcane molasses. When microbial cells are exposed to stress, stress factors will inhibit microbial growth, metabolism, and fermentation performance. The refinery final molasses after the acid-base treatment was found to contain many undissociated weak acids, which can enter the cell membrane, dissociate and release anions and protons, and the pH in the cell decreases, thus inhibiting the growth of microorganisms. By destroying the phospholipid content of the cell membrane, organic acids can increase the fluidity and permeability of the cell membrane, inhibit cell growth, change the physiological characteristics such as nutrient transport and glucose uptake, and negatively affect the activity of the membrane bound ATPase. Furan derivatives are dehydrated products of hexose and pentose, which have been proven to hinder the function of fermentation enzymes, causing physiological dysfunction of the cell membrane and inhibiting the production of metabolites (Agcam, 2022). Phenolic compounds form lignin, which can destroy membranes and are supposed to interfere with the functions of hydrophobic targets in cells. The presence of such toxic compounds can also inhibit the absorption of glucose, thus affecting cell energy metabolism and leading to a decrease in intracellular ATP levels. For the refinery final molasses treated with the ion-exchange membrane, the number of living cells growing for 6 and 12 h accounts for 96.2% (Q4) and 96.8% (Q4) of the total number of cells, respectively (Figures 3E, F). Most of the toxic substances such as colloids, melanoids, organic acids, furan derivatives, and phenols in the sugarcane molasses were removed by the ion-exchange membrane. During physiological metabolism, bacteria have resistance to substrates that reduce toxicity. Without the pressure of toxic solvents, microbial cell membranes exchange substances and regulate energy conduction in cells, directly enhancing the tolerance of microorganisms. After treatment, the pH of the sugarcane molasses was 6.5–6.8, while the intracellular pH of the *C. tyrobutyricum* was not affected, and the transmembrane pH gradient maintained cell membrane ATPase activity. Organic solvents or toxic substances entering the cell membrane will destroy the permeability and integrity of the cell membrane, and affect the function of the membrane barrier and its energy conversion.

In a word, the effects of untreated or acid-base treated refinery final molasses on microbial cells are shown in the following aspects: inhibiting cell division and growth, affecting protein synthesis, denaturing and aggregating, destroying cytoskeleton, blocking cell metabolism, and reducing cell viability which ultimately leads to cell death. In the process of *C. tyrobutyricum* fermentation, many stress factors in untreated or acid-base treated sugarcane molasses will accelerate the accumulation of ROS, resulting in oxidative stress, which causes cell structure and function damage. *C. tyrobutyricum* can spontaneously inhibit the growth rate and even die spontaneously to maintain the survival of the whole population in adverse growth environments. The effects of the ion-exchange membrane on the treatment of sugarcane molasses waste is remarkable, and its selective mechanism mainly depends on the pore size screening of the membrane, that is, when the particle size in sugarcane molasses waste that is not conducive to microbial growth is larger than the pore in the selective layer, the particle is intercepted. It is reported that the resin can also be used for molasses clarification and decolorization, which is mainly based on ion exchange and macroporous adsorption. Colloid, pigment and other stress factors diffuse from the solution to the surface of the resin through the liquid membrane, and then spread to the interior of the resin, and exchange and adsorption at the position of the active group in the resin. The treatment process is affected by three steps: liquid film diffusion, particle diffusion, and chemical reaction. In this process, there are many factors that determine the adsorption performance of the macroporous resin. The adsorption effects of the macroporous resin differ with the resin model, adsorption temperature, pH of adsorption solution, and flow rate. In addition, it is necessary to investigate the elution effect of different eluents, eluent concentrations, and eluent pH on the resin, and analyze the regeneration performance of the macroporous resin (Kang and Yu, 2018). As an emerging technology, ion-exchange membrane separation technology shows great prospects because of its outstanding advantages including its high efficiency, low energy consumption, simple process, easy integration with other technologies, environmental protection, and safety. Compared with other materials (such as macroporous resins and ceramic base membranes), ionic membranes have the advantage of small volumes, low energy consumption, and low cost. The stress factors were adsorbed by van der Waals, dipole, and hydrogen bond forces. The ion-exchange membrane with uniform pore size has excellent selectivity, and the nearly perfect cylindrical straight hole makes the fluid less resistant. In addition, the ion-exchange membrane can better overcome the balance effect of flux and retention rate, and reduce the membrane pollution resulting from the adsorption of dissolved organic matter, the production of a small amount of soluble salt by deposition or surface nucleation, and the formation of strong adhesion biofilm by deposition and breeding of microorganisms. The refinery final molasses treated with the ionic membrane can enable *C. tyrobutyricum* to maintain a relatively stable permeability and integrity of the cell membrane, and build a bridge for *C. tyrobutyricum* fermentation to produce butyric acid.

### 3.4. Adaptive evolution of *C. tyrobutyricum* in refinery final molasses with sufficient carbon sources

**FIGURE 4:**
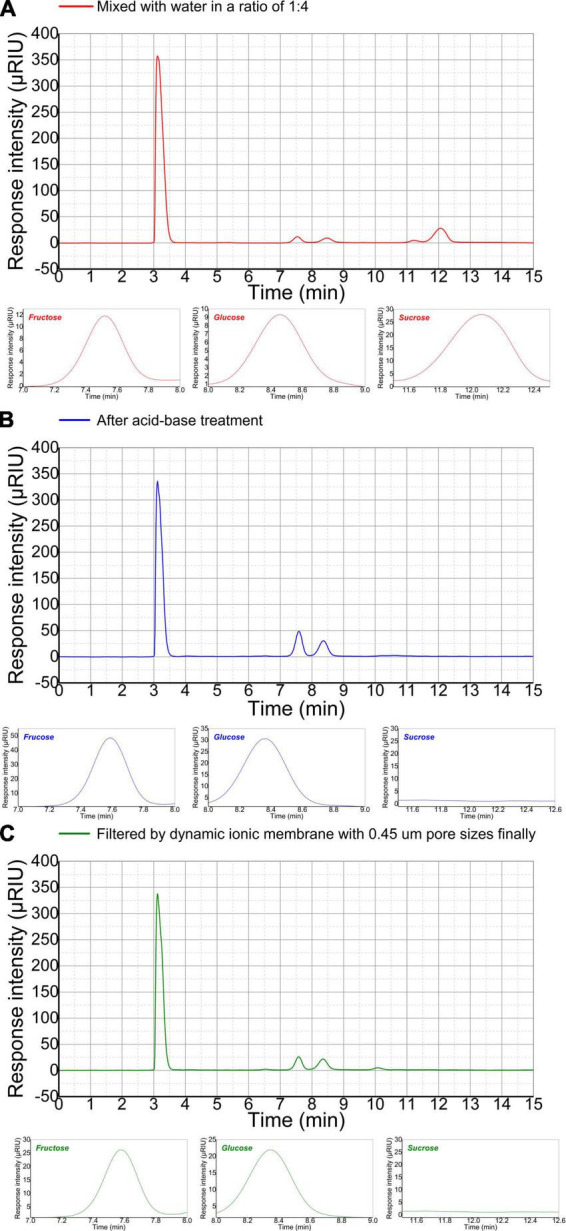
High-performance liquid chromatograms (HPLC) of the main sugars in differently treated refinery final molasses samples. Fructose appeared first at 7.53 min, glucose at 8.48 min, and sucrose at 12.06 min. The three small figures below are the enlarged images of the fructose, glucose, and sucrose peaks, respectively HPLC of refinery final molasses **(A)** diluted with water 1:4, **(B)** after acid-base treatment, and **(C)** after being filtered with 3, 1, and 0.45 μm nuclear pore membranes in turn.

The sugar composition and content of refinery final molasses is significance for butyric acid fermentation. HPLC combined with differential RID analysis show characteristics of high efficiency, rapidity, easy operation, high sensitivity, and accurate results. However, there is no relevant literature on the detection of soluble sugar in refinery final molasses by HPLC. In this manuscript, a high-performance liquid chromatography method for the determination of sugar components and content in refinery final molasses was established. Sugar molecules contain hydroxyl, aldehyde, and ketone polar groups. according to the principle of similar compatibility, non-polar chromatographic columns are used, and the six kinds of sugars have weaker interactions with the stationary phase, and the retention time in the stationary phase is shorter. This means that they quickly flows out of the chromatographic column, and the retention time is consistent, and different kinds of sugars cannot be separated. In this experiment, a polar sugar analysis column was used as the stationary phase, and water and acetonitrile were selected as the mobile phases with higher polarity. After exploration, a ratio of acetonitrile to water of 75:25 (v/v), meant that the five sugars could be well separated. The research shows that the best results were obtained when the column temperature was 30°C, the detector temperature 45°C, the sample injection volume 15.0 μL, and the flow rate 1 mL/min. According to the peak times for the five sugars, the high-performance liquid chromatograph could complete the detection of one sample after running for 30 min. It can be seen from the liquid chromatogram in Figure 4A that in the refinery final molasses that was diluted five times, the total sugar, glucose, fructose, and sucrose were 118.26, 38.03, 17.85, and 62.38 g/L, respectively. Theoretically, the acid-base treatment can convert almost all sucrose in sugarcane molasses into glucose and fructose. In the refinery final molasses after the acid-base treatment (Figure 4B), the total sugar, glucose, fructose and sucrose were 115.12, 74.95, 40.17, and 0 g/L, respectively. Furthermore, 3.14 g/L of the sugar content was lost after the acid-base treatment. The reason for this may be that calcium hydroxide as a neutralizing agent and clarifying agent was not completely clarified during the experiment, and calcium salts of approximately 200–270 mg/L remained, resulting in the loss of sugar in the refinery final molasses. The selection of appropriate separation and decolorization methods after acid-base treatment is crucial to the clarification and recycling of molasses. Studies have shown that about 60% of the macromolecular substances and pigments with a molecular size of > 3 kDa in the refinery final molasses solution can be removed by multi-stage ultrafiltration membrane filtration (Adikane and Dixit, 2009). However, approximately 12% of the sugar is lost due to the adsorption of the ultrafiltration membrane. The rejection rate of nanofiltration membrane for sucrose is > 86%, and the permeability rate of the reducing sugar is approximately 60%. Due to the concentration polarization and pollution of the feed liquid on the membrane, the nanofiltration membrane cannot perform screening without depleting sugar. Strong basic anion exchange resins can statically adsorb refinery final molasses, but its decolorization rate is inversely proportional to the sugar retention rate, which is not conducive to the experiment (Tiwari and Gaur, 2019). The ion membrane used in this study has a cut-off pore size that allows the passage of glucose and fructose with molecular weights of < 200 Da, most of the colloids, impurities, pigments, and other substances that are not conducive to the growth of microorganisms are removed, and thus, less membrane fouling occurs. In the refinery final molasses treated with ion membrane and sterilized using high temperatures and high pressures (Figure 4C), the total sugar, glucose, fructose, and sucrose were 109.94, 71.88, 38.06, and 0 g/L, respectively. The total sugar concentration loss was 5.18 g/L, and the total sugar loss rate was only 4.5%. Although the colloid in the waste cane molasses will have a certain adsorption effect on the sugar molecules, a small part of the sugar in the waste cane molasses will be taken away during the sieving process. However, by comprehensively comparing the separation technologies, the ionic membrane has been identified as the most beneficial for improving the sugar recovery rate and product quality of the refinery final molasses. The experimental results showed that maltose and lactose were not detected in the three refinery final molasses.

**FIGURE 5:**
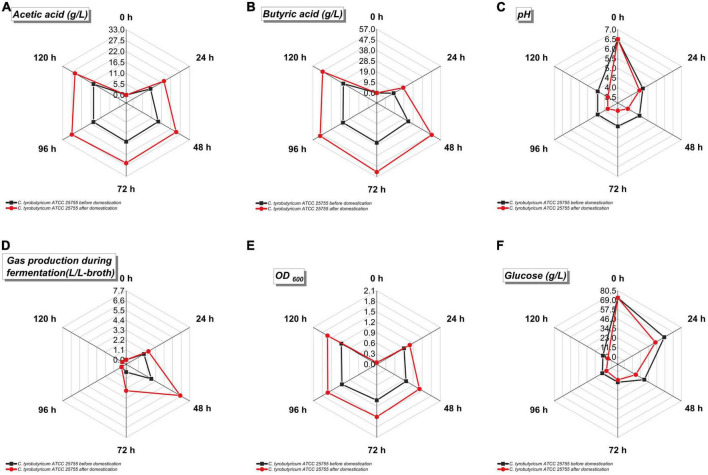
Fermentation parameters of *Clostridium tyrobutyricum* before and after acclimation in refinery final molasses treated with 0.45 μm nuclear pore membrane. The black is *C. tyrobutyricum* before domestication, and the red is *C. tyrobutyricum* after domestication. The five points on the radar chart in the figure are the sampling values at 0, 24, 48, 72, 96, and 120 h of shake flask fermentation. **(A)** Acetic acid production (g/L). **(B)** Butyric acid production (g/L). **(C)** pH value of fermentation broth. **(D)** Gas production during fermentation (L/L-broth). **(E)** Cell density of fermentation broth (OD_600_). **(F)** Glucose concentration in fermentation broth.

The sugar composition and content in the refinery final molasses after ion membrane treatment can satisfy the growth of *C. tyrobutyricum*, but for butyric acid fermentation, *C. tyrobutyricum* requires further directional adaptive evolution. Directed evolution aims to simulate Darwinian evolution process in the test tube. Through random mutation and recombination, a large number of mutations are artificially created, and selection pressure is given according to specific needs and purposes, and proteins with desired characteristics are screened out. There are four strategies for random evolution, shuffling technology, semi-rational evolution, and rational evolution (Rampelotto(Ed.), 2018). One of the outstanding features of microorganisms is their ability to rapidly adapt to various environmental conditions. This approach has been used to improve yeast strains for different biotechnological applications by performing evolutionary orientation. *Aspergillus nidulans* was used for directional adaptive evolution, and the results showed changes in its phenotype, and higher fitness of the adapted population (Dorman et al., 2018). However, this evolutionary approach has not yet been applied to generate *C. tyrobutyricum* strains that efficiently utilize refinery final molasses to produce butyric acid. If the directionality of evolution is assumed to be a definiteness or a definite change of the environment, it can be explained by the theory of natural elimination, which remains a very persuasive theory today. Therefore, in this study, using the refinery final molasses solution as the medium, the method of solid-liquid passage to screen strains was used to directionally improve the adaptability of *C. tyrobutyricum* to process refinery final molasses, and adaptive strains were screened out with high fermentation efficiency. Metabolite inhibition is a common phenomenon in the process of microbial growth. Butyric acid fermentation, as a type of organic acid fermentation, is a typical product inhibition process (Zhou et al., 2016). It was found that the inhibitory effect of metabolites was greatly reduced in strains that underwent adaptive evolution. For strains prior to adaptive evolution, butyric acid as a fermentation product would increase the metabolic burden of the bacteria at high concentrations, and the inhibitory effects of the undissociated acid production was much greater than that of the acid anion. The inhibitory effect of the butyric acid is mainly reflected *via* the decoupling effect of oxidative phosphorylation which interferes with the establishment and maintenance of the pH gradient across the cell membrane and its inhibition of cell growth. The key enzymes of protein synthesis (including PTB, BK, PTA, and AK) in bacterial metabolism, which will have corresponding inhibitory effects especially affect the growth of cells (Bellido et al., 2018). Theoretically, if the substrate carbon source is not involved in bacterial growth and metabolism to generate by-products, when the target product butyric acid is metabolized to the maximum extent, the relative yield of the butyric acid will reach 0.489 g/g (Luo et al., 2018). When the adaptively evolved strain used 71.88 g/L glucose and 38.06 g/L fructose as its carbon sources, the highest amount to of butyric acid production was 52.54 g/L, and the yield of six-carbon sugar was 0.240 g/L (Figure 5). The yield of the butyric acid increased to 0.478 g/g, which was close to the theoretical yield value of 0.489 g/g. The results demonstrate the practical value of adaptive evolution as a technical tool by which to improve the utilization of refinery final molasses by *C. tyrobutyricum* using an ion-exchange membrane-treated with sugarcane molasses.

### 3.5. Fermentation kinetics of *C. tyrobutyricum* metabolizing refinery final molasses to produce butyric acid

*Clostridium tyrobutyricum* ATCC25755 is composed of a 3.07-Mbp chromosome and a 63-kbp plasmid. It uses penta- or hexa-sugar as carbon source substrates for fermentation, and its main product is butyric acid, and it also produces acetic acid, carbon dioxide, and by-products such as hydrogen (Sjöblom et al., 2015). In the fermentation of refinery final molasses, two molecules of glyceraldehyde-3-phosphate were first generated from glucose under the action of CTK_C26180, CTK_C27660, and CTK_C01890 through the glycolysis pathway (Emdben-Meyerhof-Pamas, EMP). Then, under the action of CTK_C02250, CTK_C02260, CTK_C02280, and CTK_C02290, further redox reactions occurred to generate two molecules of pyruvate (pyruvate), as 1 mol of glucose produces 2 mol of pyruvate, accompanied by 2 mol of ATP and NADH. The generated intermediate pyruvate is catalyzed by ferritin oxidoreductase to generate acetyl coenzyme A (acetyl-CoA), and at the same time, 2 mol of CO_2_ are generated. In this process, excess NADH will be consumed to form H_2_.

The faster the growth rate of the strain, the higher the gas production during fermentation (Figures 5D, E). The growth rate of the strain after adaptive evolution in refinery final molasses was greatly improved, and the volume of the by-products H_2_ and CO_2_ was as high as 6.48 L. In order to meet the high demand for energy during bacterial growth, acetate and butyrate can be produced from the acetyl-CoA node (Figures 5A, B). Acetyl-CoA first forms acetyl phosphate (acetyl-P) under the catalysis of phosphoransacetyltransferase (PTA), and then further generates acetate under the catalysis of acetyl kinase (acetate kinase, AK). Whole-genome sequence studies have shown that *C. tyrobutyricum* does not use PTB and butyrate kinase (BK) to produce butyrate, but through redifferentiation of acetate, acetate transferase mediates butyryl-CoA (butyryl-CoA) in CTK_C06520. Under the action of butyric acid, only PTA and AK play a role in this process (Guo et al., 2020). The pH value will affect the activity of key enzymes in the bacterial metabolic pathway. Studies have shown that the pH value is 6.3–6.8, which is the most suitable for *C. tyrobutyricum* fermentation (Huang L. et al., 2011). The initial pH of the fermentation broth was 6.5 (Figure 5C). As the fermentation process progressed, more acid was produced by the strain, which lowered the pH of the fermentation broth. The PTS system is the major pathway for glucose transport in *C. tyrobutyricum*. The genome sequence analysis results of the studies indicate that CTK_C20580 is a glucose-specific PTS enzyme II gene in *C. tyrobutyricum*, which mainly takes up glucose (Calabia and Tokiwa, 2007). Therefore, this study speculates that the expression levels of PTS II enzymes and HPr in the adaptive *C. tyrobutyricum* strains increased during the growth and logarithmic growth phases, while the expression levels of PTS I gradually decreased. Based on the previous reverse transcription-PCR (RT-PCR) and proteome analysis, the cat1 (CTK_C06520) gene is the only gene directly responsible for butyrate biosynthesis, and this study speculates that the process of adaptive evolution also has a certain impact on this gene. The growth of *C. tyrobutyricum* was completely inhibited when the furfural content in the medium reached 2.5 g/L. Studies have shown that 5-hydroxymethylfurfural (5-HMF) can be generated by intramolecular dehydration of glucose under weakly acidic conditions, and the longer the autoclaving time and the higher the temperature, the higher the amount of 5-HMF generated. Short-chain dehydrogenase/reductase (SDR) and aldo-keto reductase (AKR) were shown to reduce furfural to the less toxic furfuryl alcohol in a one-step reaction with furfural and NAD(P)H as substrates (Halder et al., 2020). Compared with the original strain, the adaptively evolved *C. tyrobutyricum* significantly improved the butyric acid production rate, as well as the rate of glucose consumption, which was presumably altered by SDR and AKR in its genome, thereby improving its tolerance to furfural. In refinery final molasses, the glucose that enters the cell through active transport is mainly converted to pyruvate through the EMP pathway, while the catalysis of 6-phosphofructokinase (pfk A) and pyruvate kinase (pyk A) in the EMP pathway is an irreversible reaction. The further phosphorylation of fructose-6-phosphate to fructose-1,6-bisphosphate catalyzed by 6-phosphofructokinase is also the only rate-limiting step in the EMP pathway, so these two genes are critical for the fermentation process. The overexpression of pfkA and pykA could theoretically accelerate the EMP pathway and enhance the rate of glucose consumption in *C. tyrobutyricum*. It has been confirmed that the overexpression of pfkA and/or pykA in *C. acetobutylicum* and *Escherichia coli* can enhance intracellular NADH and ATP levels. The recombinant strain ATCC 25755/pfkA+pykA has higher glucose utilization than the original strain (Suo et al., 2018). The butyric acid production rate and the glucose consumption rate of *C. tyrobutyricum* after adaptive evolution were significantly enhanced, indicating that the adaptive *C. tyrobutyricum* with a higher intracellular ATP content could better maintain various intracellular energy-requiring metabolic activities under butyric acid stress. We speculate that this may be attributed to the adaptation evolution in refinery final molasses, which caused certain changes in the pfk A and pyk A genes of the strain. In addition, the growth of *C. tyrobutyricum* was inhibited when the glucose concentration in the medium was higher than 60 g/L during butyric acid fermentation. The adapted strains can better maintain the intracellular glucose concentration in a high-concentration glucose environment (Figure 5F). This change gives the strain the advantages of significantly accelerating the fermentation cycle, improving equipment utilization, reducing the possibility of bacterial contamination during feeding, saving resources, and reducing costs. Studies have shown that the overexpression of the Class I heat shock protein genes groESL and htpG can increase butyrate tolerance (Suo et al., 2017). In *C. tyrobutyricum*, Class I heat shock protein genes include groES, groEL, grpE, dnaK, dnaJ, and htpG. Among them, DnaK plays an important role in the process of Gram-positive bacteria responding to extracellular stress, as well as protein folding, translation, synthesis, and decomposition. For example, the *Clostridium botulinum* ΔdnaK mutant has increased sensitivity to changes in temperature, pH, and NaCl. Therefore, it is speculated that the expression of the Class I heat shock protein gene may have corresponding changes during the subculture of *C. tyrobutyricum* using refinery final molasses as the substrate.

The fermentation mechanism of anaerobic bacteria is complex, and the related research on the genome and proteomics of *C. tyrobutyricum* are scarce, and thus more research is required. In the future, further research should also be conducted to further confirm the findings presented in this manuscript. Furthermore, the mechanisms utilized by *C. tyrobutyricum* to metabolize sugar in the refinery final molasses will be analyzed in depth, to provide theoretical support to improve the production of butyric acid. However, it is certain that the ion-membrane-treated refinery final molasses can be used as the fermentation substrate for *C. tyrobutyricum*. After the adaptive evolution of *C. tyrobutyricum* for refinery final molasses, its utilization efficiency and substrate production have been greatly improved, and the adaptive strain clearly has considerable industrial fermentation potential.

## 4. Conclusion

To sum up, in order to utilize *C. tyrobutyricum* to obtain high-yield of butyric acid at low cost from refinery final molasses, The acid-base treatment method combined with ionic membrane technology was used to transform sucrose into a reducing sugar, and intercept the suspended solids, colloids, metal ions, and pigments in the solution. The established HPLC method showed that 62.38 g/L sucrose, 38.03 g/L glucose, and 17.85 g/L fructose in refinery final molasses were transformed into 74.95 g/L glucose and 40.17 g/L fructose after treatment. The ionic membrane had the advantages of causing less membrane pollution, a simple operation process, and could be conveniently cleaned, and the total sugar loss rate of the refinery final molasses filtered by the ion-exchange membrane was 4.5% after treatment. The treated refinery final molasses can meet the needs of *C. tyrobutyricum* growth tentatively, then the ordinary culture medium was replaced with the refinery final molasses to domesticate some adaptive strains at random. After seven generations of solid-liquid joint culture, a high acid producing mutant of *C. tyrobutyricum*, which could grow in refinery final molasses, was adaptively evolved. The yield of butyric acid arrived 52.54 g/L, and the yield of hexose increased from 0.240 to 0.478 g/g, which was close to the theoretical value of 0.489 g/g.

In this study, a membrane treatment technology was developed to pretreat refinery final molasses. Recycled refinery final molasses could be utilized for the growth of *C. tyrobutyricum* to produce butyric acid. The developed novel system provides a new idea for the production of butyric acid and provides some guidelines for biological resources field. The use of the waste from agro-industrial production processes to produce higher-value fermentation products increases the added value of the waste, while reducing the cost of production products, and can pay more attention to the combination of environmental friendliness. In the future, *C. tyrobutyricum* will make further use of cheap biomass to produce high value-added products, which has the potential to become a low-cost and high -benefit development model.

## Data availability statement

The original contributions presented in this study are included in this article/supplementary material, further inquiries can be directed to the corresponding authors.

## Author contributions

BW wrote the manuscript. BW and XZ conceived and designed the study. BW, XZ, and WL performed the experiments and data analyses. M-HL, DM, Q-FW, and Y-JW contributed reagents, materials, and analysis tools and performed data acquisition. M-HL, Y-JW, M-MZ, LC, SY, and BZ helped to perform the analysis with constructive discussions. XZ, XL, and DL critically revised the manuscript. XZ and DL final approved of the version to be published. All authors contributed to the discussion and comments on the manuscript.

## References

[B1] Adikane H. V. Dixit J. N. (2009). Effect of different operational conditions on the decolorization of molasses spent wash using once developed soil inoculum. *Biodegradation* 20 867–874. 10.1007/s10532-009-9274-y 19543982

[B2] Agcam E. (2022). A kinetic approach to explain hydroxymethylfurfural and furfural formations induced by Maillard, caramelization, and ascorbic acid degradation reactions in fruit juice-based mediums. *Food. Anal. Methods* 15 1286–1299.

[B3] Akhtar T. Hashmi A. S. Tayyab M. Anjum A. A. Saeed S. Ali S. (2020). Bioconversion of agricultural waste to butyric acid through solid state fermentation by Clostridium tyrobutyricum. *Waste Biomass. Valor.* 11 2067–2073. 10.1007/s12649-018-0475-7

[B4] Aung N. N. Khaing E. E. Mon Y. Y. (2022). History of sugarcane breeding, germplasm development and related research in myanmar. *Sugar. Tech.* 24 243–253. 10.1007/s12355-021-01079-y

[B5] Baroi G. N. Gavala H. N. Westermann P. Skiadas I. V. (2017). Fermentative production of butyric acid from wheat straw: economic evaluation. *Ind. Crop. Prod.* 104 68–80. 10.1016/j.indcrop.2017.04.008

[B6] Bellido C. Lucas S. González-Benito G. García-Cubero M. T. Coca M. (2018). Synergistic positive effect of organic acids on the inhibitory effect of phenolic compounds on acetone-butanol-ethanol (ABE) production. *Food Bioprod. Process.* 108 117–125. 10.1016/j.fbp.2018.02.004

[B7] Bogdanowicz K. A. Tylkowski B. Giamberini M. (2013). Preparation and characterization of light-sensitive microcapsules based on a liquid crystalline polyester. *Langmuir* 29 1601–1608. 10.1021/la3038878 23245267

[B8] Calabia B. P. Tokiwa Y. (2007). Production of D-lactic acid from sugarcane molasses, sugarcane juice and sugar beet juice by *Lactobacillus delbrueckii*. *Biotechnol. Lett.* 29 1329–1332. 10.1007/s10529-007-9408-4 17541505

[B9] Dorman C. J. Bhriain N. N. Dorman M. J. (2018). “The evolution of gene regulatory mechanisms in bacteria,” in *Grand Challenges in Biology and Biotechnology* Ed. Rampelotto S. H. (Cham: Springer), 125–152. 10.1007/978-3-319-69078-0_6

[B10] Fonseca B. C. Bortolucci J. da Silva T. M. dos Passos V. F. de Gouvêa P. F. Dinamarco T. M. (2020). Butyric acid as sole product from xylose fermentation by a non-solventogenic Clostridium beijerinckii strain under controlled pH and nutritional conditions. *Bioresour. Technol. Rep.* 10:100426. 10.1016/j.biteb.2020.100426

[B11] Ghazali N. F. Razak N. D. A. (2021). Recovery of saccharides from lignocellulosic hydrolysates using nanofiltration membranes: A review. *Food Bioprod. Process.* 126 215–233. 10.1016/j.fbp.2021.01.006

[B12] Guo S. Luo J. Yang Q. Qiang X. Feng S. Wan Y. (2019). Decoloration of molasses by ultrafiltration and nanofiltration: Unraveling the mechanisms of high sucrose retention. *Food Bioprocess. Tech.* 12 39–53. 10.1007/s11947-018-2189-z

[B13] Guo X. Fu H. Feng J. Hu J. Wang J. (2020). Direct conversion of untreated cane molasses into butyric acid by engineered *Clostridium tyrobutyricum*. *Bioresour. Technol.* 301:122764. 10.1016/j.biortech.2020.122764 31958691

[B14] Halder P. Kundu S. Patel S. Marzbali M. H. Parthasarathy R. Shah K. (2020). Furfural and levoglucosenone production from the pyrolysis of ionic liquid pre-treated sugarcane straw. *Cellulose* 28 133–151. 10.1007/s10570-020-03547-2

[B15] Huang J. Cai J. Wang J. Zhu X. Huang L. Yang S. T. (2011). Efficient production of butyric acid from Jerusalem artichoke by immobilized *Clostridium tyrobutyricum* in a fibrous-bed bioreactor. *Bioresour. Technol.* 102 3923–3926. 10.1016/j.biortech.2010.11.112 21169015

[B16] Huang L. Xiang Y. Cai J. Jiang L. Lv Z. Zhang Y. (2011). Effects of three main sugars in cane molasses on the production of butyric acid with *Clostridium tyrobutyricum*. *Korean J. Chem. Eng.* 28 2312–2315. 10.1007/s11814-011-0110-9

[B17] Ivleva E. A. Alekhin Y. V. Makarova M. A. (2020). Analysis of the features of the behavior of track membrane filters during their use. *Moscow Univ. Geol. Bull.* 75 277–285. 10.3103/S0145875220030047

[B18] Jiang L. Wang J. Liang S. Cai J. Xu Z. (2011). Control and optimization of *Clostridium tyrobutyricum* ATCC 25755 adhesion into fibrous matrix in a fibrous bed bioreactor. *Appl. Biochem. Biotechnol.* 165 98–108. 10.1007/s12010-011-9236-9 21484272

[B19] Jiang L. Wang J. Liang S. Wang X. Cen P. Xu Z. (2009). Butyric acid fermentation in a fibrous bed bioreactor with immobilized *Clostridium tyrobutyricum* from cane molasses. *Bioresour. Technol.* 100 3403–3409. 10.1016/j.biortech.2009.02.032 19297150

[B20] Kang S. Yu J. (2018). Maintenance of a highly active solid acid catalyst in sugar beet molasses for levulinic acid production. *Sugar. Tech.* 20 182–193. 10.1007/s12355-017-0543-5

[B21] Khaire K. C. Moholkar V. S. Goyal A. (2021). Bioconversion of sugarcane tops to bioethanol and other value added products: An overview. *Mater. Sci. Energy Technol.* 4 54–68. 10.1016/j.mset.2020.12.004

[B22] Li J. Liang L. Cheng J. Huang Y. Zhu M. Liang S. (2012). Extraction of pigment from sugarcane juice alcohol wastewater and evaluation of its antioxidant and free radical scavenging activities. *Food Sci. Biotechnol.* 21 1489–1496. 10.1007/s10068-012-0197-8

[B23] Li Q. L. Cui C. Wang W. (2021). Research progress on the main active components and physiological functions of sugarcane molasses and beet molasses. *Food Mach.* 37 207–211. 10.13652/j.issn.1003-5788.2021.04.038

[B24] Liu T. Zhu L. Zhu Z. Jiang L. (2020). Genome sequence analysis of *Clostridium tyrobutyricum*, a promising microbial host for human health and industrial applications. *Curr. Microbiol.* 77 3685–3694. 10.1007/s00284-020-02175-0 32888044

[B25] Luo H. Yang R. Zhao Y. Wang Z. Liu Z. Huang M. (2018). Recent advances and strategies in process and strain engineering for the production of butyric acid by microbial fermentation. *Bioresour. Technol.* 253 343–354. 10.1016/j.biortech.2018.01.007 29329775

[B26] Mahat S. B. Omar R. Idris A. Mustapa Kamal S. M. Mohd Idris A. I. (2018). Dynamic membrane applications in anaerobic and aerobic digestion for industrial wastewater: A mini review. *Food Bioprod. Process.* 112 150–168. 10.1016/j.fbp.2018.09.008

[B27] Meghana M. Shastri Y. (2020). Sustainable valorization of sugar industry waste: Status, opportunities, and challenges. *Bioresour. Technol.* 303:122929. 10.1016/j.biortech.2020.122929 32037190

[B28] Mo D. Liu J. D. Duan J. L. Yao H. J. Latif H. Cao D. L. (2014). Fabrication of different pore shapes by multi-step etching technique in ion-irradiated PET membranes. *Nucl. Instrum. Methods Phys. Res. B* 333 58–63. 10.1016/j.nimb.2014.04.011

[B29] Moore P. H. Ming R. (2011). Sugarcane breeding and biotechnology to feed the emergent sugarcane biorefinery industry. *Tropical. Plant. Biol.* 4 1–2. 10.1007/s12042-011-9075-4

[B30] Nunes L. J. R. Loureiro L. M. E. F. Sá L. C. R. Silva H. F. C. (2020). Sugarcane industry waste recovery: a case study using thermochemical conversion technologies to increase sustainability. *Appl. Sci.* 10:6481. 10.3390/app10186481

[B31] Rampelotto P. H. (Ed.) (2018). “The relevance and challenges of studying microbial evolution,” in *Grand Challenges in Biology and Biotechnology*, (Cham: Springer), 1–11. 10.1007/978-3-319-69078-0_1

[B32] Satyawali Y. Balakrishnan M. (2008). Wastewater treatment in molasses-based alcohol distilleries for COD and color removal: A review. *J. Environ. Manage.* 86 481–497. 10.1016/j.jenvman.2006.12.024 17293023

[B33] Sen K. Y. Baidurah S. (2021). Renewable biomass feedstocks for production of sustainable biodegradable polymer. *Curr. Opin. Green Sust.* 27:100412. 10.1016/j.cogsc.2020.100412

[B34] Sjöblom M. Matsakas L. Christakopoulos P. Rova U. (2015). Production of butyric acid by *Clostridium tyrobutyricum* (ATCC25755) using sweet sorghum stalks and beet molasses. *Ind. Crops. Prod.* 74 535–544. 10.1016/j.indcrop.2015.05.041

[B35] Srivastava S. (2020). “Diversification of sugar and sugarcane industry: Agro-industrial alternatives,” in *Sugar and Sugar Derivatives: Changing Consumer Preferences*, eds Mohan N. Singh P. (Berlin: Springer), 151–169. 10.1007/978-981-15-6663-9_10

[B36] Suo Y. Luo S. Zhang Y. Liao Z. Wang J. (2017). Enhanced butyric acid tolerance and production by Class I heat shock protein-overproducing *Clostridium tyrobutyricum* ATCC 25755. *J. Ind. Microbiol. Biotechnol.* 44 1145–1156. 10.1007/s10295-017-1939-7 28439766

[B37] Suo Y. Ren M. Yang X. Liao Z. Fu H. Wang J. (2018). Metabolic engineering of *Clostridium tyrobutyricum* for enhanced butyric acid production with high butyrate/acetate ratio. *Appl. Microbiol. Biotechnol.* 102 4511–4522. 10.1007/s00253-018-8954-0 29627851

[B38] Tiwari S. Gaur R. (2019). “Treatment and recycling of wastewater from distillery,” in *Applied Environmental Science and Engineering for a Sustainable Future*, Ed. Loerinczi E. (Berlin: Springer), 117–166. 10.1007/978-981-13-1468-1_5

[B39] Wei D. Liu X. Yang S. T. (2013). Butyric acid production from sugarcane bagasse hydrolysate by *Clostridium tyrobutyricum* immobilized in a fibrous-bed bioreactor. *Bioresour. Technol.* 129 553–560. 10.1016/j.biortech.2012.11.065 23270719

[B40] Zhang L. Mo D. Zhang T. D. (2018). Application of nuclear track membrane in tea filtration. *Nuclear Tech* 41:8.

[B41] Zhang S. Wang J. Jiang H. (2021). Microbial production of value-added bioproducts and enzymes from molasses, a by-product of sugar industry. *Food Chem.* 346:128860. 10.1016/j.foodchem.2020.128860 33385915

[B42] Zhou X. Yang Z. Jiang T. T. Wang S. Y. Liang J. P. Lu X. H. (2016). The acquisition of *Clostridium tyrobutyricum* mutants with improved bioproduction under acidic conditions after two rounds of heavy-ion beam irradiation. *Sci. Rep.* 6:29968. 10.1038/srep29968 27426447 PMC4947956

[B43] Zhu J. Y. Pan X. (2022). Efficient sugar production from plant biomass: current status, challenges, and future directions. *Renew. Sustain. Energy Rev.* 164:112583. 10.1016/j.rser.2022.112583

